# Vocal quality impact of thyroidectomized patients underwent inferior laryngeal nerves monitoring

**DOI:** 10.1016/j.bjorl.2025.101740

**Published:** 2025-11-26

**Authors:** Roberto Massao Takimoto, Márcio Abrahão, Felipe Gregolin Brandão, Lara Hossepian Hojaij, Onivaldo Cervantes

**Affiliations:** a Universidade Federal de São Paulo (UNIFESP), Escola Paulista de Medicina (EPM), Departamento de Otorrinolaringologia e Cirurgia de Cabeça e Pescoço, Disciplina de Cirurgia de Cabeça e Pescoço, São Paulo, SP, Brazil; b Faculdade de Ciências Médicas da Santa Casa de São Paulo (FCMSCSP), São Paulo, SP, Brazil

**Keywords:** Thyroidectomy, Dysphonia, Intraoperative neurophysiologic monitoring, Inferior laryngeal nerve, Voice quality

## Abstract

**Objective:**

The present study’s goal was to estimate the inferior laryngeal nerves monitoring impact on patient’s vocal quality after thyroidectomy in head and neck surgery training program.

**Methods:**

Eighty-five patients were evaluated, mean age 48,19 yrs., with no thyroid hormone dysfunctions. They were randomly distributed into two groups. Forty patients were operated on without nerve monitor (Group A) and other 45 with nerve monitor (Group B). The groups were controlled by gender, age, tobacco use, thyroid volume, surgery extent, post-op hypoparathyroidism, malignancy and laryngeal trauma. The patients went through perceptive evaluation and acoustic vocal analysis, before and after surgery (1st and 4th week). The same group of head and neck resident surgeons operated on all patients.

**Results:**

In perceptive evaluation, only breathiness (4th week), showed significant difference between the groups (25% in Group A vs. 6.7% in Group B, p = 0.041). However, when the patients were compared with themselves, evaluating the status before versus after surgery, none of the parameters, not even breathiness, showed any significant difference. In vocal acoustic analysis, no significant difference was observed. Fundamental frequency (Hz) in women was 202.62 (before surgery), 198.19 (1st-week) and 191.90 (4th-week) in Group A, and 193.48 (before surgery), 190.63 (1st week) and 186.81 (4th week) in Group B.

**Conclusion:**

At the present study, there was no impact, neither improvement nor worsening, in vocal quality of patients submitted to thyroidectomies with inferior laryngeal nerves monitoring.

**Level of evidence:**

Level 1.

## Highlights

• Inferior laryngeal nerves monitoring does not show impacts on residents’ performances.
• Almost 30% of all patients of the study had worsened their grade of dysphonia after surgery in perceptual analysis.

## Introduction

Goiters are known since the ancient times and the Chinese literature advocated seaweed, an important iodine source, as treatment.^1^ The first attempts on thyroid surgery probably happened in the presence of big goiters causing respiratory obstruction. One of the first reported successful case was made by Albucasis^2^ in the Xth century A.D. In the XIXth and XXth centuries, due to the work of great surgeons such as Theodore Kocher, thyroid surgery became well established with low mortality rates. From that point on, a continuous search for lower surgical complications is still being held.

One of the most fearful complications after thyroidectomy is the laryngeal nerves injury and vocal quality loss. As described by Zhang et al. (2020), the average incidence of Recurrent Laryngeal Nerve paralysis after thyroidectomy stands between 2.3% permanently and 9.8% temporary, showing a still relevant number of these complications,^3^ which could include dysphonia, aphonia, dyspnea, dysphagia and loss of safety and effectiveness in swallowing.

Many different methods of inferior laryngeal nerves preservation have been described, one of the most common is the continuous inferior laryngeal nerve monitoring with surface electrodes. There have been many described results regarding the use of this technology. Wojtczak et al. (2023) concluded that the risk factors for injuries of the inferior laryngeal nerves only contributed to vocal fold paralysis on the visualization alone cases, suggesting a safer surgery using monitorization.^4^ Furthermore, Wilhelm et al. (2023), associated the use of routine Intraoperative Neuromonitoring (IONM) with lower risk of Recurrent Laryngeal Nerve (RLN) lesion and hypoparathyroidism.^5^ Despite of that, Cirocchi et al. (2019),^6^ Cleere et al. (2022)^7^ and Karpathiotakis et al. (2022)^8^ could not establish the association between the use of IONM and less vocal cord palsy outcomes in thyroidectomies in comparison to other techniques of neural conservation. Regarding the divergent results between studies, there is still space for more studies on this topic.

Regardless of this divergency, the lack of experience of the surgeon has been previously recognized as an important risk factor for poor outcomes in thyroid surgery.^9^, ^10^, ^11^ This situation can be exemplified by surgery residents, who are still in the process of learning and polishing surgery techniques. Considering that, this study aimed to verify if the inferior laryngeal nerves intra-operative monitoring had any impact on patient’s vocal quality after thyroidectomies performed by head and neck surgery residents.

## Methods

All patients over 18-years-old that had indication for thyroidectomy, from the Head and Neck Discipline ambulatory, were invited to enroll in this study.

The exclusion criteria were patients with; previous voice disorders, previous neck surgery, previous larynx disorders (such as vocal cord palsy and tumors), previous head and neck radiation therapy, thyroid hormonal disorders and neck dissection during the thyroid surgery.

Patients were randomized into two groups. Group A had 40 patients submitted to thyroidectomies without inferior laryngeal nerves monitoring and Group B had 45 patients submitted to thyroidectomies with inferior laryngeal nerves intra-operative monitoring (NIM-Response® 2.0 Medtronic XOMED). Groups were controlled by gender, age, tobacco use, thyroid volume, surgery extent (total or partial thyroidectomy), malignancy and larynx trauma during intubation. Patients and voice therapists were blind in relation to the groups. This study was approved and reviewed by the institutional review board at the Federal University of São Paulo and all patients signed the Informed Consent.

### Surgical and anesthesiological techniques

The anesthesiologist routinely used no neuromuscular blocking agent for Group B patients. If necessary for intubation, Succinylcholine Chloride was used. The electrodes position was verified by laryngoscopy before the surgery started. All the patients were operated on by the same group of head and neck surgery residents with near supervision by the Head and Neck Discipline staff. All the procedures were made by the conventional open technique. The inferior laryngeal nerves were stimulated with 0.5–1.0 mA during the procedures.

### Laryngoscopic analysis

The larynx was assessed during breathing and during emission of the vowels /ε/ and /i/, at the patients' usual intensity and frequency.

The following were used to perform the exams: – Machida® flexible nasofibroscope, model ENT – 30PIII; – Panasonic® micro camera, model GP-AD22TA; – Iomega® ScreenPlay™ Pro HD Multimedia Player; – Mitsubishi® Diamond Scan video monitor.

The exams were recorded preoperatively, at the end of the first postoperative week and at the end of the fourth postoperative week. They were edited with images of the larynx only, so that it was impossible to identify the patients. They were recorded on a DVD-R (EMTEC 4.7 GB – Makrolon®) identified only by numbers, so that it was not possible to identify the patient or whether the exam was pre- or postoperative. The examinations were evaluated by three head and neck surgeons, independently and blindly by two examiners. The following parameters were analyzed: (1) Vocal fold mobility, classified as normal, paralysis and paresis. For analysis purposes, those with paralysis and paresis were grouped as having abnormal mobility. (2) Covering lesions such as polyps, granulomas and malignant neoplasms.

### Vocal analysis

All patients were evaluated before surgery, one-week Post-Operative (PO) and four weeks PO. Patient’s voices were recorded using the software Sound Force® 4.5. The vocal sample was /a/, /e/, /ε/, /i/, /o/ and /u/ emissions.

### Perceptual analysis

Three experienced voice therapists made the perceptual analysis. Each one individually evaluated the voice samples, and everyone was blind in relation to the groups and if the voice sample was from before or after surgery. Ten percent of the vocal samples were randomly repeated to check the reliability of each evaluator. The Kappa correlation was used to choose which evaluator had the highest reliability.

The parameters evaluated were the GRBASI scale (Grade, Roughness, Breathiness, Asthenia, Strain and Instability), modified by Dejonckere et al. (1996),^12^ from the original works of Hirano (1981).^13^ Each of these parameters were rated on a severity scale, ranging from 0 (normal), to 1 (low alteration), 2 (mid alteration) and 3 (severe alteration).

### Acoustic vocal analysis

The acoustic vocal analysis was made with the software VoxMetria® 4.0. The parameters used were:

Fundamental frequency (F0) ‒ normality range for Brazilian women is 150 up to 250 Hz, and 80 up to 150 Hz for men.

Jitter – normality range for Brazilian speakers in VoxMetria® is up to 0.6%.

Shimmer – normality range for Brazilian speakers in VoxMetria® is up to 6.5%.

Glottal-to-Noise Excitation Ratio (GNE) – normality range for Brazilian speakers in VoxMetria® is from 0.5 to 1.0.

Phonatory Deviation Diagram (PDD), is a graphic in which is considered jitter, shimmer, GNE and the Matching Waveform Coefficient (MWC).^14^ The PDD classifies voices into normal or abnormal.

### Statistical analysis

The statistical analysis was made using the software SPSS® 22 for Mac.

The Normality test was performed to verify whether a given variable was adequate to the data distribution of the Normal (Gauss) curve. This test helped choosing the test to be used, since we have parametric tests (on data that follow the Normal curve) and nonparametric tests (on data that does not follow the Normal curve).

Kolmogorov-Smirnov Normality Test for sample greater than 30 cases.

The Chi-Square test was used to verify differences in distribution of a categorized characteristic (2 or more categories) in function of another also categorized, measuring the degree of relationship between the two characteristics, in independent samples. In some cases, the Chi-Square test cannot be applied due to the low frequency observed in some classifications.

The nonparametric Mann-Whitney test was used when it was desired to compare two groups of information with a numerical measurement level and the samples were independent and it was not desired to make assumptions about the distribution of the samples analyzed.

The independent *t*-test was indicated when it was desired to compare two groups of information with a numerical measurement level, the samples were independent and it was desired to know whether, on average, the two groups were different.

The ANOVA (Analysis of Variance) test was indicated to compare three or more groups of information with a numerical measurement level, the samples were independent or paired and to know whether the groups were different in terms of averages.

For analysis purposes, a p-value was considered significant when it was less than 0.05. In order to highlight significant results, these were followed by an asterisk (*).

## Results

### Laryngoscopic analysis

There were no statistical differences between the groups in the laryngoscopies performed before surgery, in the first week after surgery or in the fourth week after surgery in any parameter.

In addition, none of the patients in the present study presented bilateral vocal fold mobility alteration.

## Discussion

Park et al.^15^ analyzed voice parameters in a retrospective review of incidence of dysphonia with 592 consecutive patients who underwent total thyroidectomies, in which 28.6% of cancer patients and 27.1% of benign lesions patients had worsened dysphonia after surgery. Side by side with this article, Iyomasa et al.^16^ in a prospective study evaluated 151 patients who underwent variated types of thyroidectomies, registering 27.8% of vocal symptoms in first postoperative that decreased to 7.2% after 6-months of surgery. Now, considering the present study, 32.5% of patients without IONM and 22.2% with IONM had worsened their grade of dysphonia in the first postoperative week while 27.5% of patients without IONM and 31.1% with IONM had worsened their grade of dysphonia in the fourth postoperative week. These results follow the general numbers of dysphonia in after thyroidectomy literature, although it should be expected recovery of the complication in the course of time. Nevertheless, the interval of 3-weeks between evaluations can be considered as insufficient to total recovery, as in Iyomasa et al. study there was a 6-month period to revaluation before the improvement of voice parameters.

Stevens et al.^17^ studied 91 patients submitted to thyroidectomies (39 with laryngeal nerves monitoring and 52 without it – groups were not randomized). They used the same nerve monitor system of the present study and stimulated the inferior laryngeal nerves with similar intensity. General and head and neck surgeons made the procedures. No training surgeon took part in this study. They evaluated the patients before surgery, one to four weeks after surgery and six months after surgery. Voice evaluation was made by perceptual analysis, acoustic analysis, laryngoscopy and the Voice Handicap Index (VHI) questionnaire. They found no significant difference in any of the parameters analyzed.

Silva et al.^18^ evaluated 308 patients submitted to thyroidectomies (100 with inferior laryngeal nerves monitor and 208 without it – groups were not randomized) one year after surgery. They used the same nerve monitor system of the present study and stimulated the inferior laryngeal nerves with 1 mA. The vocal outcomes were studied by voice questionnaire (VHI). They found no significant difference among the groups.

In addition, Wojtczak et al.^4^ evaluated the record of 711 patients that underwent thyroidectomies (1265 recurrent laryngeal nerves at risk of injury). In 257 of them (469 RLNs at risk) IONM was used, while in the other 454 patients (796 RLNs at risk) visualization alone was the method used for neural security. Both groups had no statistical demographic differences (p > 0.05). In the IONM group, the NIM-3.0 nerve monitor (Medtronic, Jacksonville, USA) with the intermittent IONM technique was used, with 0.5–1.5 mA of range. The parameter evaluated in this study was the focal fold paralysis by video or indirect laryngoscopy, preoperative, 2-days to 2-weeks postoperative and 1-year after the surgery. The results did not present significant statistical differences between the groups in the vocal fold paralysis general incidence (p > 0.05), but there were statistical differences between the groups when the patients had risk factors: the only risk factor that was statistically significant to lesions in the inferior laryngeal nerves while using IONM was the surgeon experience, while other factors were mitigated using monitorization. This shows the relevance of the present study, which proposes to evaluate the use of monitoring in the surgical training programs.

At the present study all patients were evaluated by perceptual and acoustic analysis. In the perceptual analysis (Table 1), it was found no difference in the parameters except for breathiness in the fourth week after surgery, in which Group A had 25% of patients with some breathiness against 6.7% of Group B (p < 0.041). Then these same perceptual parameters (GRBASI) were studied comparing each patient with themselves, first before surgery and then in the first and in the fourth week after surgery. At this time (Table 2), none of the parameters, not even breathiness, showed any significant difference. Another important finding concerning the perceptual analysis (Table 2), at the present study, is that almost 30% of all patients had worsen their grade of dysphonia. In the acoustic analysis, the parameters were studied by mean value (Table 3) and classified by the normality range of each parameter (Table 4). None of the acoustic parameters studied showed any significant difference between the two groups. Although the previous studies did not analyze specifically the influence of the laryngeal nerves monitoring when used by surgery residents, these results are in conformity with the literature. Also, the last of the studies shows that the inferior laryngeal nerve monitoring in use with less experienced surgeons is similar to the use of visualization alone and the experience was shown as still a factor of risk to neural lesion. These findings walk together to the results found on the current study.

**Table 1:** Perceptual analysis.

Parameters	Moment		Group A	Group B	p^a^
Grade	Before Surgery	Normal	25 (62.5%)	23 (51.1%)	0.402
		Altered	15 (37.5%)	22 (48.9%)	
	1st Week PO	Normal	14 (35%)	22 (48.9%)	0.283
		Altered	26 (65%)	23 (51.1%)	
	4th Week PO	Normal	17 (42.5%)	18 (40%)	0.990
		Altered	23 (57.5%)	27 (60%)	
Roughness	Before Surgery	Normal	27 (67.5%)	22 (48.9%)	0.130
		Altered	13 (32.5%)	23 (51.1%)	
	1st Week PO	Normal	20 (50%)	20 (44.4%)	0.768
		Altered	20 (50%)	25 (55.6%)	
	4th Week PO	Normal	22 (55%)	17 (37.8%)	0.170
		Altered	18 (45%)	28 (62.2%)	
	Before Surgery	Normal	35 (87.5%)	35 (77.8%)	0.374
		Altered	5 (12.5%)	10 (22.2%)	
Breathiness	1st Week PO	Normal	32 (80%)	42 (93.3%)	0.133
		Altered	8 (20%)	3 (6.7%)	
	4th Week PO	Normal	30 (75%)	42 (93.3%)	0.041^a^
		Altered	10 (25%)	3 (6.7%)	
Asthenia	Before Surgery	Normal	35 (87.5%)	42 (93.3%)	0.584
		Altered	5 (12.5%)	3 (6.7%)	
	1st Week PO	Normal	29 (72.5%)	40 (88.9%)	0.099
		Altered	11 (27.5%)	5 (11.1%)	
	4th Week PO	Normal	34 (85%)	41 (91.1%)	0.592
		Altered	6 (15%)	4 (8.9%)	
Strain	Before Surgery	Normal	34 (85%)	40 (88.9%)	0.834
		Altered	6 (15%)	5 (11.1%)	
	1st Week PO	Normal	34 (85%)	42 (93.3%)	0.372
		Altered	6 (15%)	3 (6.7%)	
	4th Week PO	Normal	33 (82.5%)	41 (91.1%)	0.392
		Altered	7 (17.5%)	4 (8.9%)	
Instability	Before Surgery	Normal	30 (75%)	34 (75.6%)	1.000
		Altered	10 (25%)	11 (24.4%)	
	1st Week PO	Normal	25 (62.5%)	30 (66.7%)	0.862
		Altered	15 (37.5%)	15 (33.3%)	
	4th Week PO	Normal	26 (65%)	31 (68.9%)	0.881
		Altered	14 (35%)	14 (31.1%)	

a Chi-Square test.

**Table 2:** Perceptual analysis comparing each patient status before surgery with themselves after surgery.

Parameters	Moment		Group A	Group B	p^a^
Grade	1st Week PO	Stable	27 (67.5%)	35 (77.8%)	0.412
		Worse	13 (32.5%)	10 (22.2%)	
	4th Week PO	Stable	29 (72.5%)	31 (68.9%)	0.900
		Worse	11 (27.5%)	14 (31.1%)	
Roughness	1st Week PO	Stable	33 (82.5%)	35 (77.8%)	0.587
		Worse	7 (17.5%)	10 (22.2%)	
	4th Week PO	Stable	33 (82.5%)	33 (73.3%)	0.452
		Worse	7 (17.5%)	12 (26.7%)	
Breathiness	1st Week PO	Stable	37 (92.5%)	45 (100%)	0.200
		Worse	3 (7.5%)	0	
	4th Week PO	Stable	36 (90%)	42 (93.3%)	0.871
		Worse	4 (10%)	3 (6.7%)	
Asthenia	1st Week PO	Stable	34 (85%)	42 (93.3%)	0.372
		Worse	6 (15%)	3 (6.7%)	
	4th Week PO	Stable	38 (95%)	44 (97.8%)	0.917
		Worse	2 (5%)	1 (2.2%)	
Strain	1st Week PO	Stable	37 (92.5%)	43 (95.6%)	0.892
		Worse	3 (7.5%)	2 (4.4%)	
	4th Week PO	Stable	34 (85%)	42 (93.3%)	0.372
		Worse	6 (15%)	3 (6.7%)	
Instability	1st Week PO	Stable	33 (82.5%)	36 (80%)	0.987
		Worse	7 (17.5%)	9 (20%)	
	4th Week PO	Stable	32 (80%)	36 (80%)	1.000
		Worse	8 (20%)	9 (20%)	

a Chi-Square test.

**Table 3:** Acoustical parameters.

Parameters	Moment		Group A	Group B	p^a^
F0 women (Hz)	Before Surgery	Mean	202.62	193.48	0.773
		SD	29.64	42.41	
	1st Week PO	Mean	198.19	190.63	
		SD	35.47	47.49	
	4th Week PO	Mean	191.90	186.81	
		SD	34.53	41.63	
Jitter (%)	Before Surgery	Mean	0.30	0.29	0.949
		SD	0.42	0.29	
	1st Week PO	Mean	0.47	0.53	
		SD	0.90	1.42	
	4th Week PO	Mean	0.20	0.24	
		SD	0.11	0.30	
Shimmer (%)	Before Surgery	Mean	3.92	4.39	0.8136
		SD	2.36	3.74	
	1st Week PO	Mean	4.51	4.80	
		SD	3.72	5.67	
	4th Week PO	Mean	3.88	3.76	
		SD	2.35	1.85	
GNE	Before Surgery	Mean	0.84	0.85	0.920
		SD	0.15	0.15	
	1st Week PO	Mean	0.81	0.83	
		SD	0.17	0.14	
	4th Week PO	Mean	0.82	0.84	
		SD	0.17	0.16	

a ANOVA test.

**Table 4:** Acoustical parameters and the PDD, classified by the normality range.

Parameters	Moment		Group A	Group B	p^a^
F0 (Hz)	Before Surgery	Normal	34 (85%)	37 (82.22)	0.959
		Altered	6 (15%)	8 (17.78)	
	1st Week PO	Normal	34 (85%)	33 (73.3%)	0.295
		Altered	6 (15%)	12 (26.7%)	
	4th Week PO	Normal	34 (85%)	36 (80%)	0.750
		Altered	6 (15%)	9 (20%)	
Jitter (%)	Before Surgery	Normal	36 (90%)	40 (88.9%)	1.000
		Altered	4 (10%)	5 (11.1%)	
	1st Week PO	Normal	35 (87.5%)	40 (88.9%)	1.000
		Altered	5 (12.5%)	5 (11.1%)	
	4th Week PO	Normal	40 (100%)	41 (91.1%)	0.156
		Altered	0	4 (8.9%)	
Shimmer (%)	Before Surgery	Normal	37 (92.5%)	39 (86.7%)	0.604
		Altered	3 (7.5%)	6 (13.3%)	
	1st Week PO	Normal	35 (87.5%)	40 (88.9%)	1.000
		Altered	5 (12.5%)	5 (11.1%)	
	4th Week PO	Normal	37 (92.5%)	40 (88.9%)	0.844
		Altered	3 (7.5%)	5 (11.1%)	
GNE	Before Surgery	Normal	39 (97.5%)	43 (95.6%)	1.000
		Altered	1 (2.5%)	2 (4.4%)	
	1st Week PO	Normal	37 (92.5%)	44 (97.8%)	0.526
		Altered	3 (7.5%)	1 (2.2%)	
	4th Week PO	Normal	35 (87.5%)	42 (93.3%)	0.584
		Altered	5 (12.5%)	3 (6.7%)	
PDD	Before Surgery	Normal	36 (90%)	40 (88.9%)	1.000
		Altered	4 (10%)	5 (11.1%)	
	1st Week PO	Normal	33 (82.5%)	40 (88.9%)	0.595
		Altered	7 (17.5%)	5 (11.1%)	
	4th Week PO	Normal	36 (90%)	40 (88.9%)	1.000
		Altered	4 (10%)	5 (11.1%)	

a Chi-Square test.

On the other hand, the use of IONM in thyroidectomies seems to allow less dissection of the tissue, what can protect the parathyroids. Still, there can be increased protection to bilateral vocal fold paralysis and the routine use of this technique can increase the surgeon's feel safety,^19^ taking to increased patient safety. These last statements should be discussed in upcoming studies.

## Conclusion

In conclusion, the present study does not show impact of the use of IONM of the inferior laryngeal nerve in vocal quality of patients submitted to thyroidectomies performed by a surgical training program group.

## ORCID ID

Márcio Abrahão: 0000-0003-0184-1437

Lara Hossepian Hojaij: 0009-0001-6324-4585

Onivaldo Cervantes: 0009-0007-6057-6756

## Funding

This work was supported by The National Research Council (CNPq); Sociedade Beneficente Israelita Brasileira Albert Einstein (SBIBAE).

## Declaration of competing interest

The authors declare no conflicts of interest.
